# A Comparative Study Between Intravenous Esmolol and Oral Clonidine in Attenuating Hyperdynamic Cardiovascular Response to Laryngoscopy and Endotracheal Intubation

**DOI:** 10.7759/cureus.64584

**Published:** 2024-07-15

**Authors:** V N K Srinivas Mudiganti, Brig TVSP Murthy, Sneha Kakara, Mudiganti Raja Sri Jaya Iswarya, Pratap R, Amol Prakash Singam, Abhishek Jain

**Affiliations:** 1 Critical Care Medicine, Jawaharlal Nehru Medical College, Datta Meghe Institute of Higher Education and Research, Acharya Vinoba Bhave Rural Hospital (AVBRH), Wardha, IND; 2 Anesthesiology, GSL Medical College and General Hospital, Rajahmundry, IND; 3 Anesthesiology and Critical Care Medicine, GSL Medical College, Rajahmundry, IND; 4 Anesthesiology and Critical Care Medicine, Command Hospital, Armed Forces Medical College (AFMC), Pune, IND; 5 Anesthesiology, Army Hospital Research and Referral (R and R), Delhi, IND; 6 Cardiology, Krishna Institute of Medical Sciences (KIMS), Rajahmundry, IND; 7 Radiology, Konaseema Institute of Medical Sciences and Research Foundation, Amalapuram, IND; 8 Critical Care Medicine, Jawaharlal Nehru Medical College, Datta Meghe Institute of Higher Education and Research, Wardha, IND

**Keywords:** pressor response, hemodynamic changes, endotracheal intubation, clonidine, esmolol

## Abstract

Background

In today's era of anesthesia, balanced anesthesia is the main basis of patient care and pain management. Of all the medications given during general anesthesia, premedication, induction agents, and muscle relaxants play a major role in keeping the hemodynamics properly under control. When laryngoscopy is performed to intubate, a pain stimulus will be generated, leading to a rise in blood pressure and heart rate. This stimulus can be avoided without any complications if appropriate premedication is given to the patient at the appropriate dosage. In this research, we compare the influence of injection esmolol and oral clonidine during the time of induction as premedications to suppress the hemodynamic response.

Material and methods

In a prospective randomized controlled trial, 90 patients were divided into three groups: Group E (esmolol) received 2 mg/kg IV esmolol diluted in 0.9% NS two minutes pre-anesthesia; Group C (clonidine) received oral clonidine 4 mcg/kg 90 minutes pre-anesthesia; and Group P (placebo) received IV normal saline and oral water. Blood pressure, heart rate, and mean arterial pressure were measured at baseline and seven subsequent time points.

Results

The study compared systolic blood pressure (SBP), mean arterial pressure (MAP), and diastolic blood pressure (DBP) changes over seven minutes in three groups, clonidine (Group C), placebo (Group P), and esmolol (Group E). At one minute, Group E showed a consistent MAP decrease from 95.21 mmHg to 85.92 mmHg, while Group C and Group P exhibited fluctuating trends. DBP decreased across all groups, with Group P ending highest (77.7 mmHg) and Group C lowest (66.8 mmHg). Group E's SBP decreased steadily from 126.2 mmHg to 118.0 mmHg, Group C decreased from 128 mmHg to 116.1 mmHg, and Group P showed more erratic fluctuations in SBP, DBP, and MAP.

Conclusion

These findings suggest that intravenous esmolol shows a good hemodynamic response having superior control over heart rate and getting the pressure under control quickly without major drop compared with the clonidine and placebo groups.

## Introduction

At the time of general anesthesia during the induction phase, many hemodynamic changes take place. The changes can be seen during laryngoscopy and endotracheal intubation [1]. At the time of laryngoscopy on visualization of the epiglottis, there is a reflex rise in heart rate and blood pressure [2]. In young individuals, this sympathetic response may not cause many complications, but in elderly patients with comorbidities and coronary artery disease (CAD), this leads to severe complications and adverse effects such as cardiac arrhythmia, myocardial infarction, and cerebrovascular accidents [3]. Laryngoscopy and intubation hold a major role during general anesthesia and critical care. Immediately after laryngoscopy, there will be a rise in systolic and diastolic blood pressure of about 25-30 mmHg. It will peak after one to two minutes and gradually decrease in normal patients. High systolic blood pressure (SBP) and diastolic blood pressure (SBP) and heart rate, however, may not go down and may even worsen conditions in individuals with comorbidities such as CAD [4]. The main reason for the reflex sympathetic response is laryngoscopy, compared to endotracheal intubation and circulating catecholamines [5]. Techniques such as deepening the plane of anesthesia [6] and drugs such as nitroglycerine [7], beta-blockers [8], calcium channel blockers [9], gabapentin [10], clonidine [11], and opioids such as fentanyl [12] and remifentanil [13] were used as pre-medications to reduce the sympathetic response and control the hemodynamic response during laryngoscopy. Using a technique or a drug depends on the patient’s clinical condition and the comorbidities [14]. Balanced anesthesia and resuscitative measures require laryngoscopy and endotracheal intubation. At the time of laryngoscopy, the scope touches the base of the tongue and causes hemodynamic changes. The lingual branch of the mandibular nerve supplies the front two-thirds of the tongue, the glossopharyngeal nerve supplies the posterior one-third, and the hypoglossal nerve provides motor innervation [15].

Esmolol is a beta-1-selective antagonist. It is a short-acting drug with 8-10 minutes of half-life and a volume of distribution of 2 L per kilogram. The drug is eliminated by plasma hydrolysis by plasma esterase. It has little sympathomimetic activity and is given intravenously [16]. A useful antihypertensive drug is clonidine hydrochloride, which lowers blood pressure and heart rate while maintaining peripheral resistance and cardiac output. It has little effect on renal blood flow or glomerular filtration rate. Renin release is decreased through a central action. Some of the cardiovascular effects of clonidine result from its central agonistic properties. There is a centrally mediated reduction in sympathetic activity due to its effect on post-synaptic receptors in the vasomotor center of the medulla [17]. This study sets out to tackle a common challenge in anesthesia: how to best keep patients safe during laryngoscopy and endotracheal intubation. These procedures often trigger significant variations in hemodynamics such as SBP, DBP, mean arterial pressure (MAP), and heart rate, and while there are many ways to try to manage these responses, there is not one clear winner. Hence, we are comparing two different medications, intravenous esmolol and oral clonidine, to see which one does a better job of keeping patients stable during these moments. Ultimately, our goal is to find an approach that ensures the highest level of safety and improves outcomes for patients undergoing these procedures.

## Materials and methods

Aim

To assess the effect on hemodynamic parameters after administering intravenous esmolol or oral clonidine during the time of induction in general anesthesia as premedication.

Study setting and design

The study was conducted on 90 adult patients in the Department of Anesthesiology of GSL Medical College and General Hospital in Rajahmundry. It employed a prospective randomized controlled trial, covering the period from March 2017 to March 2018.

Sample size and data analysis

The sample size was calculated based on the mean difference for MAP according to Campagni et al.'s [18] article, which is 5 mmHg, with a pooled standard deviation of 8.5. The assumptions included a Z alpha value of 1.96 at a 5% significance level and a power (1-beta) of 99% equating to 2.34. The required sample size was 28 per group, accounting for a 10% dropout rate (= 2). We have included 30 samples in each group. Statistical analysis was done by Student's t-test, where the sample mean is used to estimate the population means and the corresponding p-value was obtained (p < 0.05 was considered significant, p < 0.01 was considered highly significant, and p > 0.05 was considered statistically insignificant).

Participants

After obtaining written informed consent, a total of 90 participants were selected who were posted for general surgical, gynaecological, and orthopaedics surgeries. They were of both sexes. Their age ranged from 18 to 50 years, and their weight ranged from 40 to 75 kg. The patients were carefully selected according to the American Society of Anaesthesiologists classification and were in grades I and II. They were randomly stratified as follows: 30 participants were assigned to the esmolol group, labeled Group E; 30 participants to the clonidine group, labeled Group C; and 30 participants to the placebo group, labeled Group P. Group E patients were given an injection of esmolol (2 mg/kg IV) diluted in 0.9% NS two minutes before induction of anesthesia. They were also given 10 mL of water ninety minutes before giving the induction agent. Ninety minutes before giving the induction agent, Group C patients were given tablets of clonidine (4 micrograms per kg) orally in 10 mL of water. They were also given 10 mL of 0.9% NS for two minutes before giving the induction agent. Group P patients were given both 10 mL of normal saline intravenously two minutes before and 10 mL of water 90 minutes before induction of anesthesia.

Data collection

In the preoperative assessment, all the patient's history was taken, which also included drug allergies. A detailed general physical examination was done. Preoperative tests such as hemoglobin, total leucocyte count, serum creatinine, serum electrolytes, liver function tests, 2D echocardiography, electrocardiography, and chest X-ray were advised. The day before the surgery, all patients received a tablet of Lorazepam (2 mg) orally at 10 p.m. On the day of surgery, a tablet of lorazepam (2 mg) was given orally two hours before, and an injection of glycopyrrolate (0.2 mg) was given intravenously 20 minutes before giving the induction agent. Every participant was preoxygenated for five minutes. Injection thiopentone is used as an induction agent, and succinylcholine is used as a muscle relaxant as a part of rapid sequence intubation. Electrocardiography (ECG), pulse oximetry, heart rate, and blood pressure were monitored continuously. Methods for inducing anesthesia were utilized, which included the administration of succinylcholine and thiopentone intravenously. Continuous monitoring of non-invasive blood pressure, oxygen saturation (SpO_2_), pulse rate, and ECG was carried out during the induction procedure. We measured each patient's heart rate, SBP, DBP, mean arterial blood pressure, and SPO_2_ before administering the premedication. These parameters were taken as initial values. After 90 minutes of clonidine and two minutes of esmolol, induction agents were given. After confirmation of complete muscle relaxation, a laryngoscopy was performed by a senior anesthesiologist, and then intubation was done in a single attempt. Altered anatomy of the mouth and neck, particularly dental structure that might pose a problem to smooth and quick intubation-patients having such anticipated intubation problems, was excluded from the study. At baseline time 0 (zero), followed by the first, second, third, fourth, fifth, sixth, and seventh minutes of interval after laryngoscopy, SBP, DBP, heart rate, and SpO_2_ are noted. On one occasion, there was a sudden drop in blood pressure to less than 78/56 mmHg necessitating attention and was managed accordingly. This case was excluded from the study. On one occasion, intubation could not be performed on a single attempt, and prolonged laryngoscopy was needed, which required the insertion of a laryngeal mask airway, so they were also excluded from the study.

Ethical considerations

The institutional ethical committee of GSL Medical College and General Hospital has approved, and informed consent was obtained from each patient. The Institutional Ethics Committee (IEC) and Institutional Review Board (IRB) reference numbers are GSLMC/RC: 353-EC/353/09/16, dated 09/09/2016.

## Results

Baseline blood pressure in Group E (SBP of 122 mmHg and SBP of 72 mmHg with a MAP of 91.2), Group C (SBP of 124 mmHg and DBP of 77 mmHg with a MAP of 91.8), and Group P (SBP of 127 mmHg and DBP of 74 mmHg with a MAP of 91.7) All groups experienced a peak increase in SBP at one minute. At three minutes, Group P had the highest SBP, while Group E had the lowest. Compared to the control group, the clonidine and esmolol groups showed a minimal rise in SBP one minute after intubation. With clonidine and esmolol, SBP returned to the initial value within two minutes after laryngoscopy and dropped below the basal level at five and seven minutes, which was statistically significant (p < 0.05). In Group P, SBP returned to the basal level more than seven minutes after intubation (Table 1).

**Table 1:** Comparision of systolic blood pressure among the control and study groups Group E - Esmolol group, Group C - Clonidine group, Group P - Placebo group

Group/Time	Group E	Group C	Group P	F value	P value
0 min	122 ± 4.59	124 ± 7.11	127 ± 11.12	2.919	0.0593
1 min	126.2 ± 5.11	128 ± 6.59	131.1 ± 8.27	4.08	0.0216(sig.)
2 min	125 ± 4.89	124.2 ± 5.12	132 ± 9.11	12.44	<0.001(sig.)
3 min	133.9 ± 8.11	136.3 ± 9.12	147.4 ± 12.1	15.806	<0.001(sig.)
4 min	131.4 ± 7.11	131.2 ± 6.77	142.2 ± 11.1	20.126	<0.001(sig.)
5 min	125.2 ± 4.88	125.6 ± 4.52	138.3 ± 11.1	29.834	<0.001(sig.)
6 min	122.3 ± 4.56	120.7 ± 4.89	136 ± 9.11	49.845	<0.001(sig.)
7 min	118 ± 4.11	116.1 ± 4.24	131.6 ± 7.22	75.175	<0.001(sig.)

There was a peak increase in DBP from a baseline (Group E = 72 mmHg, Group C = 77 mmHg, and Group P = 74 mmHg) just after laryngoscopy and intubation (time: three minutes). This peak increase was more in the placebo group and less in the esmolol group (Table 2). Patients of the esmolol group showed a significant rise just after laryngoscopy and intubation at one minute and after four minutes, came closer to baseline at five minutes, and significantly lower than baseline at six and seven minutes of laryngoscopy and intubation, which are statistically significant. Patients in the clonidine group had a significant rise in DBP just after laryngoscopy and intubation of one minute, but not so at four and five minutes after laryngoscopy and intubation. However, at six and seven, DBP was lower than baseline, and this was statistically significant. Group P patients showed a significant rise in DBP from baseline at three and four minutes and came to baseline after seven minutes.

**Table 2:** Comparison of changes in diastolic pressure in control and study groups Group E - Esmolol group, Group C - Clonidine group, Group P - Placebo group

Group/Time	Group E	Group C	Group P	F value	P value
0 min	72 ± 6.92	77 ± 8.67	74 ± 7.98	3.052	0.0523
1 min	76.5 ± 5.11	81.4 ± 9.02	78.1 ± 8.21	3.213	0.045(sig.)
2 min	79.6 ± 5.76	82.3 ± 8.027	82.7 ± 7.21	1.711	0.1868
3 min	82.5 ± 5.24	86.3 ± 7.89	89.6 ± 8.2	7.23	0.0012(sig.)
4 min	78.6 ± 4.67	70.7 ± 3.12	85 ± 6	68.369	<0.001(sig.)
5 min	75.2 ± 4.98	69.1 ± 4.11	83.7 ± 7.65	48.289	<0.001(sig.)
6 min	71.3 ± 4.11	67.8 ± 3.67	80.7 ± 4.66	76.91	<0.001(sig.)
7 min	69.6 ± 4.32	66.8 ± 3.65	77.7 ± 4.41	56.071	<0.001(sig.)

There was a peak increase in MAP from baseline (Group E = 91.21 mmHg, Group C = 91.8 mmHg, and Group P = 91.7 mmHg) immediately after laryngoscopy and intubation at three minutes. This increase was most pronounced in the clonidine group and minimal in the esmolol group. Compared to the control Group P patients, both clonidine and esmolol caused a decrease in MAP at five and seven minutes after intubation, with a significance level of p = 0.05 (Table 3) Patients of the esmolol group also showed a significant rise in MAP just after one minute after laryngoscopy and intubation and three and four minutes; however, it was closer to baseline after five minutes. They showed a significant reduction in MAP at six and seven minutes after laryngoscopy and intubation. Patients in the clonidine group had a significant rise in MAP just after one minute and three and four minutes after laryngoscopy and intubation; however, it was closer to baseline after six minutes. Group P patients had a significant rise in MAP at three, four, and six minutes after laryngoscopy and intubation, but it was closer to baseline at seven minutes after laryngoscopy and intubation, which is statistically significant.

**Table 3:** Comparison of changes in mean arterial blood pressure in control and study groups Group E - Esmolol group, Group C - Clonidine group, Group P - Placebo group

Group/Time	Group E	Group C	Group P	F value	P value
0 min	92.2 ± 5.8	92.8 ± 5.01	92.7 ± 6.78	3.011	0.0544
1 min	95.21 ± 5.85	95.8 ± 5.67	95.7 ± 5.73	0.13	0.878
2 min	95.5 ± 5	99.1 ± 7	96.5 ± 5.01	3.136	0.0484(sig.)
3 min	101.14 ± 7.14	108.9 ± 12.12	102.4 ± 9.01	5.593	0.0052(sig.)
4 min	97.3 ± 6.11	104.1 ± 8.12	98.1 ± 8.01	7.425	0.0011(sig.)
5 min	94.3 ± 6.13	101.9 ± 7.21	94 ± 6.11	14.21	<0.001(sig.)
6 min	89.8 ± 5.25	99.1 ± 6.21	91.8 ± 5.78	21.668	<0.001(sig.)
7 min	85.92 ± 5.27	95.7 ± 6.89	87.1 ± 5.71	23.783	<0.001(sig.)

There was an increase in pulse rate from baseline (Group E = 68 beats /min, Group C = 79 beats/min, and Group P = 74 beats/min) in all the groups. At three minutes, the pulse rate was the highest in Group P (92.9 ± 5.39) and was the lowest in the esmolol group (78.4). At four minutes, the increase in pulse rate was more in Group C (90.6) and minimal in the esmolol group (75.5). Thereafter, the pulse rate gradually decreased. In Group P, the pulse rate returned to the baseline value after seven minutes (Table 4).

**Table 4:** Comparison of the heart rate in control and study groups Group E - Esmolol group, Group C - Clonidine group, Group P - Placebo group

Group/Time	Group E	Group C	Group P	F value	P value
0 min	68 ± 3.67	79 ± 5.66	74 ± 4.11	43.752	<0.001(sig.)
1 min	70.7 ± 3.69	81.6 ± 5.87	76 ± 4.28	40.275	<0.001(sig.)
2 min	74.5 ± 3.78	86.7 ± 6.11	81.8 ± 5.28	42.669	<0.001(sig.)
3 min	78.4 ± 4.02	92.3 ± 6.89	92.9 ± 6.28	58.768	<0.001(sig.)
4 min	75.5 ± 3.99	90.6 ± 5.90	88.3 ± 5.78	70.801	<0.001(sig.)
5 min	71.8 ± 4.02	88.4 ± 5.95	84.8 ± 5.98	78.591	<0.001(sig.)
6 min	68.2 ± 3.68	84.5 ± 5.99	80 ± 5.22	83.183	<0.001(sig.)
7 min	64.4 ± 3.02	82.2 ± 5.85	76.5 ± 4.88	110.727	<0.001(sig.)

**Figure 1:**
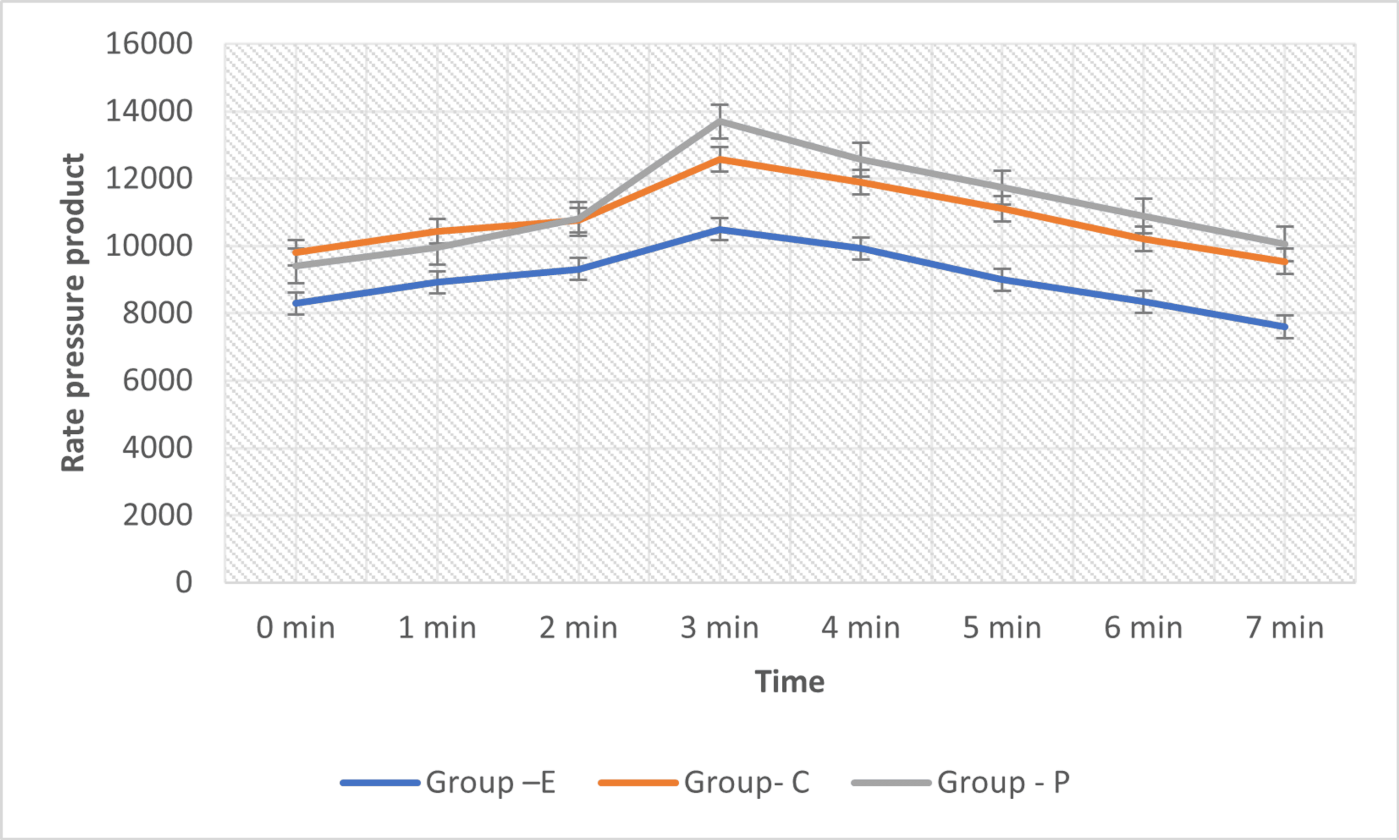
Product of mean heart rate and systolic blood pressure F value = 7.9421, p value = 0.0027 Since p value = 0.0027 < p = 0.05, it is significant.

A peak increase in the rate pressure product (RPP) was observed just after laryngoscopy and intubation and three minutes after intubation in all the Groups (Figure 1).

SBP and DBP, heart rate, and MAP were noted at baseline, followed by first, second, third, fourth, fifth, sixth, and seventh. The research compared changes in SBP, MAP, and DBP, over seven minutes among three groups: clonidine (Group C), placebo (Group P), and esmolol (Group E). Analysis revealed varying patterns among the groups. Group E exhibited a consistent decrease in MAP from 92.2 ± 5.8 mmHg to 85.92 ± 5.27 mmHg, while Group C and Group P demonstrated fluctuating trends. DBP decreased in all groups, with Group P showing the highest final value (77.7 ± 4.41 mmHg) at seven minutes and Group C exhibiting the lowest final value (66.8 ± 3.65 mmHg). SBP decreased steadily in Group E from 122 ± 4.59 mmHg to 118 ± 4.11 mmHg, while clonidine from 124 ± 7.11 mmHg to 116.1 ± 4.24 mmHg with the highest MAP 108.9 ± 12.12 at time 3 compared with Group E with a MAP 102.4 ± 9.01 mmHg at three minutes, and placebo group displayed more erratic fluctuations in SBP, DBP, and MAP. These findings suggest distinct cardiovascular responses to the experimental condition, potentially indicating a therapeutic effect.

The analysis shows significant differences in the mean heart rates between Group E, Group C, and Group P over the seven time points. Group C generally has the highest mean heart rates, followed by Group P, with Group E having the lowest mean heart rates across the measured time points. The statistical analysis confirms that these differences are not due to random chance.

## Discussion

A study done by Singh evaluated the consequences of oral versus intravenous clonidine premedication on blood pressure and heart rate responses to endotracheal intubation in 100 normotensive patients aged 18-45 years undergoing elective surgery. Patients were given clonidine (3 µg/kg) either orally for 30 minutes or intravenously for 15 minutes before induction. Measurements showed that IV clonidine significantly reduced heart rate, SBP and DBP, and rate pressure product more effectively than oral clonidine. Post-intubation increases in these parameters were also less pronounced in the IV group. By 10-15 minutes post-laryngoscopy, all values returned to baseline. The study concluded that IV clonidine better attenuates the hemodynamic stress response without adverse effects [19].

This study done by Arora et al. found that the use of intravenous clonidine reduces hemodynamic responses to laryngoscopy and intubation in 90 female patients with breast cancer. All the patients were divided into three groups: a control group and two groups receiving clonidine at 1.0 μg/kg and 2.0 μg/kg. Both doses of clonidine significantly reduced heart rate, SBP and DBP, and MAP compared to the control. The higher dose (2.0 μg/kg) caused adverse effects such as hypotension and sedation, which were not seen with the lower dose. The study concluded that 1.0 μg/kg of clonidine combined with fentanyl is an effective and safe method for attenuating hemodynamic stress during these procedures [20].

In a double-blind clinical trial with 96 patients done by Montazeri et al., Group G (800 mg gabapentin) and Group C (0.3 mg clonidine) showed significantly decreased HR and RPP at five, 10, and 15 minutes post-intubation (p < 0.05). Group G had significantly lower SAP, DAP, MAP, and RPP post-intubation compared to Group P (placebo) (p < 0.05). No significant differences were found between Group C and Group P in these parameters [21].

In a double-blind, placebo-controlled study done by Shrestha et al., 60 surgical patients received either lignocaine (1.5 mg/kg), esmolol (1.5 mg/kg), or normal saline before laryngoscopy and tracheal intubation. Esmolol significantly attenuated the increase in DBP and MAP compared to lignocaine and the control groups (p < 0.05). SBP changes were not significant among the groups. The heart rate increase was significantly lower in the esmolol group compared to both the lignocaine and control groups (p < 0.05) [22].

In the present study, 90 adult patients from both sexes were allocated into three groups. Group P served as the control; they were given 5 mL of rose syrup orally, 90 minutes before induction, and 10 mL of normal saline, intravenously, two minutes before induction. Group C and Group E were pre-treated with clonidine and esmolol, respectively. In Group C, patients were given oral clonidine 4 mg/kg in tablet form, dissolved in 5 mL of rose syrup, given 90 minutes before induction, and normal saline (10 mL), which was given intravenously two minutes before induction, whereas in Group E patients, an injection of esmolol of 2 mcg/kg made to 10 mL was given, intravenously, two minutes before induction, and 5 mL of rose syrup was also given 90 minutes before induction in the same patients. In all the groups, patients were between 18 and 50 years old. The mean age was 32.85 ± 11.93 in Group C, 37.15 ± 10.1 in Group E, and 35.25 ± 11.47 in Group P. Among the total 90 cases, 58 were female, and 32 were male. All of them were in good nutritional status and free from systemic diseases. There was an increase in pulse rate (Table 4) in all the groups of patients just after laryngoscopy and intubation and one minute after intubation (three and four minutes). At three minutes, the pulse rate was the highest in Group P (92.9 ± 5.39) and was the lowest in the esmolol group (78.4). In Group C, it was 92.3 ± 3.32. When compared to the basal value, this rise was more significant in all the groups (p < 0.01). At four minutes, the increase in pulse rate was the highest in control Group C (90.6) and the lowest in the esmolol group (75.5). Thereafter, the pulse rate gradually decreased. In Group P, the pulse rate returned to the baseline value 10 minutes after intubation (seven minutes). In Group E, it returned to the baseline value two minutes after intubation, and at seven minutes, it went below the basal level, which is statistically significant (p < 0.01).

Just after laryngoscopy and intubation, there was a peak increase in SBP in all the groups. This increase was the highest in Group P (147.4 ± 7.57) and was the lowest in Group E (133.9 ± 9.07). When compared to the control group of patients, both Group C and Group E showed a minimal but statistically insignificant increase in SBP at one minute after laryngoscopy. With clonidine and esmolol, SBP returned to the basal level within two minutes after intubation and fell below the basal level at five and seven minutes, which is statistically significant (p < 0.05). In Group P, SBP returned to the basal level more than seven minutes after intubation (Table 1).

There was a peak increase in DBP just after laryngoscopy and intubation (time: three minutes). This peak increase was the highest in Group P (89.6 ± 4.03) and the lowest in the esmolol group (82.5 ± 5.44).

When compared to the control group of patients, clonidine produced no significant change at one and two minutes after intubation, whereas esmolol produced no significant change at two minutes after intubation. However, there was a significant decrease in DBP at five and seven minutes after intubation in both Group C and Group E patients (p < 0.05).

There was a peak increase in MAP just after laryngoscopy and intubation (time: three minutes). This increase was the highest in Group C (108.9 ± 3.79) and was the lowest in the esmolol group (101.14 ± 6.28). When compared to control (Group P) patients, both clonidine and esmolol produced a significant decrease in MAP at five and seven minutes after intubation (p < 0.05) (Table 3).

A peak increase in RPP was observed just after laryngoscopy and intubation [time 3] in all the groups. This increase was highest in Group P (36.63%) and was lowest in the Esmolol Group (15.58%). This rise was highly notable in all the groups [p<0.01]. 

When compared to the control group of patients, Clonidine and Esmolol produced no significant change at 2 minutes after intubation. At 7 minutes after intubation, there was a statistically significant decrease in RPP in both the clonidine and esmolol groups (p < 0.05), but not so in the control group (Figure 1).

Oral clonidine could not control the heart rate, but it produced significant control of SBP, DBP, and MAP. Moreover, it produced a statistically significant fall in pressure starting five minutes after intubation, which is not desired.

Intravenous esmolol controlled the heart in a better way. SBP and MAP throughout the study period were comparable to those of the clonidine group. Though there was a significant drop in pressure between five and seven minutes after intubation, it was not to the extent of clonidine.

The study demonstrated that, while both clonidine and esmolol were effective in controlling SBP, SBP, and MAP post-intubation, esmolol showed superior control over heart rate compared to clonidine. Both medications helped return blood pressure to baseline levels more quickly than the control group, with esmolol achieving this earlier than clonidine. Notably, esmolol produced a more stable cardiovascular response without the significant drop in pressures observed with clonidine.

The stimulation of oropharyngeal structures during laryngoscopy is a significant contributor to the hemodynamic stress response observed during tracheal intubation. This process can lead to tachycardia, hypertension, and elevated catecholamine levels, which pose a potential risk of life-threatening complications, particularly in individuals vulnerable to cardiovascular issues. Analyzing the different data obtained from this study, it was found that esmolol reduced cardiovascular responses during laryngoscopy and intubation. The action of the drug is easily controllable and reversible. It seems that esmolol is a very selective and appropriate answer to the short-time pressure response to laryngoscopy and endotracheal intubation.

Limitations

Our study has certain limitations since we were unable to examine the changes in the neuroendocrine responses to laryngoscopy and intubation between the two medicines because we did not monitor the serum levels of stress markers such as cortisol and catecholamines during the procedures. Additionally, the study design primarily focused on short-term outcomes immediately following laryngoscopy and intubation, without assessing long-term effects or patient outcomes beyond the immediate perioperative period. As the resources were limited we used non-invasive blood pressure rather than continuous invasive blood pressure monitoring, we could not calculate the sedation score. Future research with large sample sizes, longer follow-up periods, and control of confounding factors is warranted to further elucidate the comparative efficacy and safety profiles of intravenous esmolol and other drugs in similar clinical contexts.

## Conclusions

Based on the results of the study, intravenous esmolol emerges as the superior attenuator compared to the other drugs under investigation in mitigating the cardiovascular responses to laryngoscopy and intubation. As such, IV esmolol could be considered the preferred option, especially in scenarios necessitating emergency anesthesia for patients with unstable cardiovascular conditions. This finding underscores the potential clinical significance of esmolol in managing such situations, highlighting its efficacy and suitability for use in critical medical interventions.
